# Final-year pharmacy undergraduate students’ career intention and its influencing factors: a questionnaire study in northwest China

**DOI:** 10.1186/s12909-020-02342-8

**Published:** 2020-11-04

**Authors:** Tianqi Zhang, Lingui Li, Ying Bian

**Affiliations:** 1 Institute of Chinese Medical Sciences & State Key Laboratory of Quality Research in Chinese Medicine, University of Macau, Avenida da Universidade, Taipa, Macau, China; 2 College of Management, Ningxia Medical University, 1160 Shengli Street, Yinchuan, Ningxia Province China

**Keywords:** Pharmacy student, Career intention, Employment guidance, China

## Abstract

**Background:**

Career intention is closely related to the distribution of university graduates across sectors in pharmacy-related field. The aim of the study was to describe career intention and identify its influencing factors among final-year pharmacy undergraduate students in China.

**Methods:**

A questionnaire study on demographic characteristics, educational situation, family background, occupational value and career intention was conducted among final-year pharmacy undergraduate students at three universities in northwest China. Exploratory factor analysis was used to identify the constructs of occupational value. Multinominal logistic regression was adopted to analyse the factors influencing career intention.

**Results:**

Among the 275 student participants, 62.6% intended to work in public medical institutions (rural: 44.4%; urban: 18.2%), 26.5% aimed to work in the pharmaceutical industry, 6.5% wanted to work in other sectors in the pharmacy-related field, and 4.4% planned to work in other fields. Their gender, father’s education level, monthly household income per capita, whose opinions were considered most during job selection, the self-gratification factor of occupational value, and employment guidance had significant impacts on students’ career intentions.

**Conclusion:**

In the Chinese background, the career intention of more than half of the undergraduate pharmacy students was to work in public medical institutions. The career intentions of the overall participants were mainly determined by their gender, family background as well as psychological self-gratification, and they could also be influenced by employment guidance.

**Supplementary Information:**

The online version contains supplementary material available at 10.1186/s12909-020-02342-8.

## Background

Due to the rapidly developing pharmaceutical industry and the constantly changing demand for pharmacy practice, there has been a call for a higher-quality pharmacy workforce [1]. Aside from increasing the quantity, the improvement of the workforce distribution among different sectors in the pharmacy-related field is directly related to the efficiency of pharmaceutical human resource allocation and employment equity. On this basis, as the future workforce, the career choices of pharmacy students will influence pharmaceutical workforce distribution upstream, when they are employed after graduation. Since employment is a bilateral selection process between the student and the employer, employers might have a desire to know about students’ career intentions and its formation in order to select the right person for the right position. Moreover, it is of great importance for educators and policy makers to comprehend this information, to help students form appropriate career intentions before making their career choices.

There is not yet a unified definition for ‘career intention’. In this research, career intention is used to indicate a person’s inclination to pursue a specific career; ‘intention’ is generalised as the most important and immediate predictor of a behaviour by several psychological theories [2]. In other studies, career aspiration, career goal, career preference, and future career choice were expressions used to represent a similar idea. Regardless of the discrepancies between the intention and outcome, in general situations, career choice was also adopted to discuss this issue, which is the outcome of the job search. However, in real situations, it is acknowledged that due to limited competence or opportunities, a student may make a career choice differing from his/her career intention. This current research focused on career intention rather than career choice.

There have been a series of studies concerning pharmacy students’ inclination to pursue specific careers, career intention, and the dynamics of the formation of career intentions among students in BPharm and PharmD programmes. These studies were mainly conducted in North America (USA [3]), Europe (the UK [4, 5], Poland [6]), Africa (Ethiopia [7], Nigeria [8], Sierra Leone [9]), Oceania (Australia [10], New Zealand [11]), the Middle East (Kuwait [12], Jordan [13]), Southeast Asia (Malaysia [14]), and East Asia (Korea [15], Japan [16]). In general, these studies showed that pharmacy students’ career intentions were influenced by a variety of factors, which could be generally categorised as intrinsic motivation, extrinsic motivation, interpersonal factors, and professional capacity or self-awareness of one’s own professional capacity.

However, differences have also been observed among different regions in the world. For instance, studies have shown that students of Asian origin or who were educated in Asia tended to prioritise economic earnings and social status, or the potentials, which might be explained by cultural priorities [5, 17, 18]. There are also differences in the healthcare systems and labour markets for health workers among countries. Specifically, in China, pharmacists working in community pharmacies might have lower wages and social status than those working in hospital pharmacies. This may impact pharmacy students’ job selection.

As home to one-fifth of the world’s population, China continuously experiences healthcare reform. The demand for the optimisation of the pharmacy workforce is more urgent than that of the physician and nurse workforce; in particular, there is an insufficient workforce of pharmacists [19]. The density of pharmacists in China was 2.6 per 10,000 people in 2010. Although it rose to 3.17 in 2018 [19, 20], China ranked 47 among the 75 countries that submitted official data concerning this figure to the WHO. Moreover, a large proportion of Chinese pharmacists are fulfilling traditional dispensing and delivery roles but are incapable of providing qualified pharmaceutical care. Therefore, China needs more improvements in the pharmacist workforce [21].

The census conducted by the Chinese Ministry of Education showed that there were 359 educational pharmacy programmes provided by the higher medical institutions in the whole nation in 2015, and 29,125 students were enrolled that year. Due to the late introduction of the clinical pharmacist role in China, most educational pharmacy programmes in Chinese higher medical institutions are academia- or industry-oriented [22]. The majority of these institutions provide a Bachelor of Science (BS) in pharmacy for undergraduate students.

After graduation, students who pass a national entrance examination for postgraduate study could pursue master’s degrees. The others flow into the labour market, bolstering the country’s pharmaceutical workforce. A large proportion of these students work in public medical institutions to start a career as a hospital pharmacist with a licence certificate exemption, as for newly graduated students a licence certificate would normally be mandatory after entry into the workforce. Additionally, due to the dual rural-urban social structure in China, the salaries and working conditions of rural and urban public medical institutions are quite distinct from one another, geographic priority is a general phenomenon. In contrast with the public medical institutions, obstacles existed to employment in private medical institutions because of the common requirement for licence certificate, which demands several years of pre-requisite work experience. Among non-medical sectors, industry is another main flowing direction, providing a variety of positions with renumeration ranging from the worst to the best levels in this field. While positions in public departments and education institutions for newly undergraduate students are limited, some of them will leave the pharmacy-related field and search for better career development in other fields.

As students’ career intentions are their motivations for job selection and is one of the core factors to predict how they make career choices that determines their distribution in the labour market, researchers of pharmacy education have conducted a series of studies on pharmacy students’ career intentions. Nonetheless, there is insufficient research on this subject in China. Although some researchers have conducted career intention studies among undergraduates of all majors or majors other than pharmacy [23], the situation of Chinese pharmacy students is still unclear. To the authors’ knowledge, studies providing brief descriptions of BS pharmacy students’ career intentions existed, however, they were mainly conducted in one university and few of them developed extensive analysis [24, 25].

It was shown that pharmacy students have different career perspectives and psychological statuses than students in other health-related majors [4]. Due to the differences in the social environment between Confucian and Western cultures [26], Chinese pharmacy students might have their own characteristics that are different from those of pharmacy students in other countries. The aim of this research was to understand career intentions and its influencing factors among undergraduate pharmacy students in a moderately developed area of China; this result may provide useful information to educators, policy makers, international exchange students, and employers, including multinational companies.

## Methods

### Participants and procedure

A questionnaire study was conducted in the pharmacy department of three universities in Xi’an, the capital of Shaanxi Province, China. The Shaanxi Province is a moderately developed province in northwest China. In 2016, Shaanxi Province ranked 15th among all 31 provinces in mainland China in the regional annual gross domestic product (GDP), and Xi’an City’s annual GDP per capita was 66,800 *yuan* (2016 Chinese national annual GDP per capita: 53,680 *yuan*) [27]. There are seven universities and colleges that provide a bachelor’s degree in pharmacy in this province. Three are public, three are private, and one is affiliated with the military. This research was conducted at the three public universities: Xi’an Jiaotong University, Xi’an Medical University, and Shaanxi University of Chinese Medicine. In January 2016, the questionnaires were distributed to the three universities’ final-year pharmacy students when they attended briefing sessions about graduation. These briefing sessions were compulsory for all final-year pharmacy students. Since career intention is formed in the earlier years of undergraduate education, examining students’ career intention in their final year of learning allowed for the identification of the comprehensive effects of influencing factors. Two other factors that might have interfered with the ability to determine students’ true career intentions were considered: the Chinese National Entrance Examination for Postgraduate (NEEP) and students’ job search progress. To the majority of students who want to pursue postgraduate study, going through NEEP is compulsory. And the small part of students being recommended for postgraduate study were settled down before this examination. The timing of the questionnaire survey was specifically selected to minimize the influence of both the NEEP and the job search; the surveys were administered several days after the NEEP was completed but before the marks were given. At that time, most of the students had not yet signed an employment contract. Therefore, the students had formed their career intentions but did not make a choice for job selection or postgraduate study. On this basis, those who planned to enter the labour market after graduation were included in this survey, while those who might pursue a higher-level degree or a gap year were not. The students could decide whether to participate in this survey according to their own situations, including their self-prediction of NEEP marks. The questionnaire survey was anonymous, and ethical approval was obtained from Xi’an Medical University. All participants were informed of the aim of the study and were told that their information would be used only for academic research. Written informed consent was obtained from all of them. They were provided with a hardcopy of the questionnaire and required to complete it independently during the brief session. No incentives were provided.

### Questionnaire

Based on other studies on the career intentions of pharmacy students worldwide or students of other related majors in China, the potential influencing factors on career intention were divided into the following categories: demographic status [10], educational situation [3, 28], family background [23], and occupational value [29, 30]. Educational situation could also indicate one’s professional capacity, family background could represent interpersonal relationships, and occupational value included both intrinsic motivation and extrinsic motivation. Specifically, family background was used to represent students’ interpersonal relationships because, in China, family is considered the most important foundation for the nourishment of one’s interpersonal skills. On this basis, the questionnaire was designed with five sections. The first section collected demographic information, including gender, age, and region of origin (rural or urban). The second section concerned students’ educational situation during undergraduate study, including the study record (whether the student was in the top 30%), academic experience (whether the student had laboratory training), medicinal training experience (whether the student had an internship in a medical institution), and employment guidance (whether the student had received employment guidance). The third section collected family background data, including father’s education level, mother’s education level, monthly household income per capita, and autonomy of job selection (whose opinions were considered most during job selection). The fourth section presented items with a 5-point Likert-like scale to assess occupational value. The preliminary version of the scale contained 23 items, including 13 items extracted or adapted from a published scale and its revised version of occupational value among Chinese undergraduate students of all majors [29, 30], and 5 items from another related scale utilised among undergraduate students of medicine-related majors, as well as 5 self-made items tailored to the social development and the specialty of pharmacy education. After the pilot study, in total 5 items were removed, thus a final version with 18 items was obtained. The fifth section included one question on career intention, with options including village clinic, township medical centre, county hospital, prefectural hospital, provincial hospital, private medical institute, pharmaceutical industry, official department on pharmaceutical affairs, community pharmacy, educational institute, and other fields. Options that were irrelevant to undergraduate students’ job selection, such as academic track or gap year, were not provided. Participants were asked to pick the most ideal answer for all the questions; only one answer was permissible. The pilot study was conducted with individuals who had been final-year pharmacy undergraduate students at Xi’an Medical University during the previous year. Revisions were made after it, and feedback was adopted before implementing the main survey.

### Statistical analysis

All data collected by the questionnaire study were manually input into a Microsoft Excel 2013 spreadsheet. IBM SPSS version 21.0 was utilised to perform data screening and statistical analysis. Descriptive statistics were developed to show the diversity of career intentions among different subgroups. Exploratory factor analysis (EFA) was employed to determine the underlying constructs of occupational value. A multinominal logistic regression model was then built to reveal the factors influencing students’ career intentions. The test standard was set at a two-sided *p*-value of 0.05.

## Results

### Demographic information

There were a total of 405 final-year pharmacy students at the three universities; 276 returned questionnaires, and 275 returned valid questionnaires. The response rate was 68.1%. The demographic characteristics of these 275 participants are shown in Table 1. The average age of all participants was 22.6 years (range: 20–25 years). Among them, 82.5% were female. More than half of the participants were from rural areas (69.8%), and the remaining participants were from urban areas (30.2%).

**Table 1:** Descriptive analysis of career intentions (*N* = 275)

Variables		N (%)^a^	Pharmaceutical industry (%)^b^	Rural public medical institution (%)^b^	Urban public hospital (%)^b^	Other sectors in the pharmacy-related field (%)^b^	Other fields (%)^b^
**Demographic Information**							
Gender	Male	48 (**17.5**)	14 (29.2)	21 (43.8)	4 (8.3)	3 (6.3)	6 (12.5)
	Female	227 (**82.5)**	59 (26.0)	101 (44.5)	46 (20.3)	15 (6.6)	6 (2.6)
Region of origin	Rural	192 (**69.8**)	49 (25.5)	91 (47.4)	32 (16.7)	13 (6.8)	7 (3.6)
	Urban	83 (**30.2**)	24 (28.9)	31 (37.3)	18 (21.7)	5 (6.0)	5 (6.0)
**Educational Situation**							
Study record	Top 30%	92 (**33.5**)	29 (31.5)	34 (37.0)	16 (17.4)	8 (8.7)	5 (5.4)
	Other	183 (**66.5**)	44 (24.0)	88 (48.1)	34 (18.6)	10 (5.5)	7 (3.8)
Laboratory training	Yes	274 (**99.6**)	73 (26.6)	122 (44.5)	50 (18.2)	17 (6.2)	12 (4.4)
	No	1 (**0.4)**	0 (0.0)	0 (0.0)	0 (0.0)	1 (100.0)	0 (0.0)
Internship in a medical institution	Yes	102 (**37.1**)	24 (23.5)	45 (44.1)	25 (24.5)	6 (5.9)	2 (2.0)
	No	173 (**62.9**)	49 (28.3)	77 (44.5)	25 (14.5)	12 (6.9)	10 (5.8)
Employment guidance	Yes	206 (**74.9**)	57 (27.7)	93 (45.1)	37 (18.0)	15 (7.3)	4 (1.9)
	No	69 (**25.1)**	16 (23.2)	29 (42.0)	13 (18.8)	3 (4.3)	8 (11.6)
**Family Background**							
Father’s education level	Compulsory	165 (**60.0**)	40 (24.2)	82 (49.7)	31 (18.8)	5 (3.0)	7 (4.2)
	Above	110 (**40.0**)	33 (30.0)	40 (36.4)	19 (17.3)	13 (11.8)	5 (4.5)
Mother’s education level	Compulsory	195 (**70.9**)	47 (24.1)	96 (49.2)	34 (17.4)	11 (5.6)	7 (3.6)
	Above	80 (**29.1**)	26 (32.5)	26 (32.5)	16 (20.0)	7 (8.8)	5 (6.3)
Monthly household income per capita (*yuan*)	Low (~ 999)	103 (**37.5**)	13 (12.6)	66 (64.1)	13 (12.6)	6 (5.8)	5 (4.9)
	Middle (1000 ~ 1999)	86 (**31.3**)	28 (32.6)	24 (27.9)	27 (31.4)	3 (3.5)	4 (4.7)
	High (2000~)	86 (**31.3**)	32 (37.2)	32 (37.2)	10 (11.6)	9 (10.5)	3 (3.5)
Whose opinions were considered most during job selection	Others	57 (**20.7**)	9 (15.8)	24 (42.1)	16 (28.1)	4 (7.1)	4 (7.0)
	Parents	101 (**36.7**)	20 (19.8)	52 (51.5)	19 (18.8)	6 (5.9)	4 (4.0)
	Own	117 (**42.5**)	44 (37.6)	46 (39.3)	15 (12.8)	8 (6.8)	4 (3.4)
Total (**%**)^a^		275 (**100.0**)	73 (**26.5**)	122 (**44.4**)	50 (**18.2**)	18 (**6.5**)	12 (**4.4**)

^a^The denominator was the total sample size

^b^The denominator was the sample size of the subgroup represented in the row

### Career intention

Career intention was divided into five categories based on a comprehensive consideration of socioeconomic status, nature of the work, and entry requirements: rural public medical institutions (village clinic, township medical centre, or county hospital), urban public hospital (prefectural hospital or provincial hospital), pharmaceutical industry, other sectors in pharmacy-related field (private medical institution, educational institute, or official department of pharmaceutical affairs), and other fields. Among all participants, 62.6% intended to work in a public hospital/medical institution (rural: 44.4%; urban: 18.2%). Another 26.5% intended to work in the pharmaceutical industry, and 6.5% aimed to work in other sectors in the pharmacy-related field. The remaining 4.4% of the participants wanted to work in other fields. The distribution of career intentions among all subgroups is shown in Table 1.

### Factor analysis of occupational value

The Cronbach’s *α* of the total 18 items of occupational value was 0.921, and it decreased to 0.897 after 4 items (I7-I9, I18) were removed due to their cross loadings. The criterion for removal was that the loading of the specific item was larger than 0.4 for more than one factor. For factor analysis, a principal component analysis of the remaining 14 items was conducted. Finally, based on the minimal eigenvalue criterion of 1.0 and equamax rotation, three factors were extracted. The Cronbach’s *α* values were 0.897, 0.784, and 0.796 for Factors 1, 2, and 3, respectively. As shown in Table 2, according to the content of the items for each factor, Factor 1 was generated as the Job Factor (I1-I6). Factor 2 was generated as the Self-gratification Factor (I14-I17). Factor 3 was generated as the Development Factor (I10-I13). The three factors explained 65.35% of the total variance. The Kaiser-Meyer-Olkin value was 0.862, indicating that factor analysis was justifiable for this scale. The Bartlett test (*χ*^2^ = 2011.202, *p*<0.001) indicated the absence of the identity matrix, implying the data was adequate for EFA. The score of each factor was calculated for each case according to the score matrix. The functions were F_i_ = Z_i1_*I_1_ + Z_i2_*I_2_ + … + Z_i14_*I_14_ (F_i_ is the factor score, I is the item score, and Z_i_ is the score coefficient; i = 1, 2, 3).

**Table 2:** Rotated factor matrix of occupational value

Items	Code	Factor		
		Job Factor	Self-gratification Factor	Development Factor
Scale of the institution	I1	**0.828**	0.131	0.205
Economic performance of the institution	I2	**0.804**	0.258	0.190
Popularity of the institution	I3	**0.719**	0.423	−0.035
Social image of the institution	I4	**0.735**	0.347	0.103
Labour intensity	I5	**0.801**	−0.035	0.303
Wages and benefits	I6	**0.684**	0.240	0.171
Promotion opportunities	I10	0.341	0.115	**0.673**
Interpersonal relationships in the workplace	I11	0.043	0.378	**0.721**
Training and self-development opportunities	I12	0.117	0.220	**0.803**
Job-major match	I13	0.110	0.121	**0.764**
Perspective of the work	I14	0.037	**0.678**	0.256
Power of the work	I15	0.192	**0.779**	0.131
Significance of the work	I16	0.237	**0.748**	0.286
Interest in the work	I17	0.320	**0.664**	0.175

### Multinominal logistic regression

In the multinominal logistic analysis, career intention was set as the dependent variable and participants who intended to work in the pharmaceutical industry were set as the reference group. All factors related to demographic information (gender, region of origin), educational situation (study record, laboratory training, internship, and employment guidance), family background (father’s education level, mother’s education level, monthly household income per capita, whose opinions were considered most during job selection), and occupational value (Job Factor, Development Factor, and Self-gratification Factor) were first evaluated in the univariate analysis. As shown in Table 3, gender, employment guidance, father’s education level, monthly household income per capita, whose opinions were considered most during job selection, and the Self-gratification Factor of occupational value showed significant influences on the dependent variable. These six variables were put into the logistic model as independent variables, and dummy variables were set for the nominal variables. The results showed that all these variables had significant impacts on students’ career intentions. Students who had received employment guidance were less likely to work in the other fields than in the pharmaceutical industry (OR = 0.142, 95% CI: 0.034–0.591, *p*<0.01). Those whose fathers had received only the nine-year compulsory education were less likely to work in other sectors of the pharmacy-related field (OR = 0.296, 95% CI: 0.080–0.902, *p*<0.05). Those with a low family income (monthly household income per capita: < 1000 *yuan*) were more likely to work in rural public medical institutions (OR = 5.213, 95% CI: 2.301–11.810, *p*<0.001). Those with a middle-level family income (monthly household income per capital: 1000–1999 *yuan*) were more likely to work in urban public hospitals (OR = 2.880, 95% CI: 1.021–8.121, *p*<0.05). Those who considered their parents’ opinions most during job selection were more likely to work in public hospitals and medical institutions (rural: OR = 3.417, 95% CI: 1.585–7.366, *p*<0.01; urban: OR = 3.949, 95% CI: 1.473–10.589, *p* < 0.01). Those who considered others’ opinions most were more likely to work in public hospitals and medical institutions (rural: OR = 2.593, 95% CI: 1.007–6.681, *p*<0.05; urban: OR = 6.926, 95% CI: 2.308–20.779, *p <* 0.01). Male students were more likely to work in other fields (OR = 4.782, 95% CI: 1.163–20.404, *p* < 0.05). The Self-gratification Factor was positively associated with working in urban public hospitals (OR = 2.096, 95% CI: 1.323–3.321, *p* < 0.01), as shown in Table 4.

**Table 3:** Univariate analysis

Variables	−2 Log Likelihood	DF	*P*
**Gender**	**43.083**	**4**	0.033^*^
Area of origin	37.406	4	0.534
Study Record	38.839	4	0.360
Laboratory training	25.599	4	0.239
Internship in a medical institution	40.574	4	0.162
**Employment guidance**	**43.908**	**4**	0.034^*^
**Father’s education level**	**45.699**	**4**	0.024^*^
Mother’s education level	41.411	4	0.130
**Monthly household income per capita**	**89.668**	**8**	< 0.001^***^
**Whose opinions were considered most during job selection**	**64.011**	**8**	0.027^*^
Job factor	706.474	4	0.593
**Self-gratification Factor**	**706.474**	**4**	0.001^**^
Development Factor	701.787	4	0.321

^*^P < 0.05; ^**^P < 0.01; ^***^P < 0.001

**Table 4:** Influencing factors of career intention according to the multinomial logistic regression analysis

Variables^a^	Rural public medical institution			Urban public hospital			Other sectors in the pharmacy-related field			Other fields		
	OR	95% CI	*P*	OR	95% CI	*P*	OR	95% CI	*P*	OR	95% CI	*P*
Self-gratification Factor	1.140	0.816–1.592	0.443	**2.096**	**1.323–3.321**	0.002^**^	0.851	0.472–1.534	0.592	1.260	0.665–2.388	0.478
Employment guidance												
Yes	0.984	0.459–2.017	0.966	0.829	0.325–2.112	0.694	2.141	0.486–9.440	0.315	**0.142**	**0.034–0.591**	0.007^**^
No **(*Ref*)**			–			–			–			–
Father’s education level												
Compulsory	1.854	0.937–3.667	0.076	1.323	0.555–3.155	0.527	**0.296**	**0.080–0.902**	0.033^*^	1.426	0.311–6.533	0.648
Above **(*Ref*)**			–			–			–			–
Monthly household income per capita^b^												
**Low**	**5.213**	**2.301–11.810**	< 0.001^***^	2.699	0.870–8.369	0.086	2.106	0.581–7.629	0.257	5.003	0.837–29.898	0.078
Middle	1.042	0.465–2.336	0.920	**2.880**	**1.021–8.121**	0.046^*^	0.441	0.100–1.939	0.279	2.994	0.502–17.884	0.228
High **(*Ref*)**			–			–			–			–
Whose opinions were considered most during job selection												
**Others**	**2.593**	**1.007–6.681**	0.048^*^	**6.926**	**2.308–20.779**	0.001^**^	2.820	0.589–13.512	0.195	5.000	0.794–31.466	0.086
**Parents**	**3.417**	**1.585–7.366**	0.002^**^	**3.949**	**1.473–10.589**	0.006^**^	0.914	0.252–3.320	0.891	5.660	0.944–33.947	0.058
Oneself **(*Ref*)**			–			–			–			–
Gender												
Male	0.996	0.434–2.283	0.992	0.382	0.109–1.331	0.131	0.684	0.145–3.235	0.632	**4.782**	**1.163–20.404**	0.030^*^
Female ***(Ref)***												

^*^P < 0.05; ^**^P < 0.01; ^***^P < 0.001; *Ref*: reference of the independent variable

^a^The reference group of the dependent variable was the pharmaceutical industry

^b^Monthly household income per capita: low level (< 1000 *yuan*); middle level (1000–1999 *yuan*); high level (≥ 2000 *yuan*)

## Discussion

This research explored the career intentions of undergraduate pharmacy students who were about to start their professional careers. Most of the participants were approximately 22 years old, and had enrolled in universities in 2012, after passing the Chinese National College Entrance Examination in the final year of their secondary education. None was married. Because of the continuous, seamless learning process from high school to university, it was hardly for most undergraduate students to have formal working experiences. Moreover, pursuing a BS in pharmacy is time-consuming. It includes knowledge learning, laboratory training, and even clinical training. Thus, students have to spend more time on school learning and have comparatively limited time to take part in extracurricular activities or other kinds of social engagement. Therefore, undergraduate pharmacy students had finite individual social resources beyond familial and university commitments. Concerning the abovementioned aspects of age, marital status, working or internship experience and their learning process, there is high homogeneity among pharmacy students in all Chinese tertiary education institutions. This could, to some extent, be a reflection of the overall situation in China.

This study first showed the differences in career intentions among different subgroups of Chinese pharmacy undergraduate students. There were similarities and dissimilarities between this study and other related studies worldwide. In most previous studies, gender was found to have a significant influence on pharmacy students’ career intentions [15, 16]. In this research, the situation was the same. The results showed that male students were more likely to transition to non-pharmacy fields than female students, while they were less likely to work in urban public hospitals. In China, the dual rural-urban societal structure seemed to be on the foremost considerations in job selection. For men who intend to stay in urban areas for better living standards and more development opportunities, working in other non-pharmacy fields might provide more potential for better economic returns than working in public hospitals. This finding revealed that male pharmacy students were more economically oriented than the female at the beginning of their professional lives. In comparison with males, female pharmacy students were less inclined to go to non-pharmacy fields, even though both genders had a similar proportion of the top 30% of students ranked by academic performance (male: 31.3%; female: 33.9%). This result coincided with research in two other East Asian countries, Japan and Korea [15, 16]. Moreover, the regression model also supported the notion that gender was a significant influencing factor for career intention. The potential influence of this inclination on the increasing number of women in the pharmacy-related field is worth further attention.

The analysis of influencing factors showed that students prioritised their self-gratification needs, whereas two other factors of occupational value, the Job Factor and Development Factor, showed no significant influence on career intention. In the job search, self-gratification needs could be attributed to intrinsic motivation, while the need for a job could be attributed to extrinsic needs, and development needs could be attributed to both intrinsic and extrinsic needs. This result echoed those of other studies which showed that interest or motivation was one of the strongest reasons for students to develop a career intention for pharmacy-related profession [10], which could be viewed as an externalization of occupational value. However, pharmacy students in northwest China seemed to primarily consider intrinsic needs, which was distinct from the views that asserted Asian students or students of Asian origin value economic returns more than intrinsic factors [5]. Moreover, this result was different from the study in Japan, where pharmacy students ranked development opportunities as the most important factor [16]. This result was also divergent from the results showing that Malaysian Chinese students primarily valued opportunities for learning and development [14]. This phenomenon might be because Chinese students’ intrinsic needs were repressed by competitive pressure from continuous years of study from middle school through university. Thus, as a form of psychological compensation, they prioritised individual self-gratification over the job conditions, economic returns, and career development.

This research also showed that three of the five influencing factors could be attributed to family background. This finding corresponded with those of studies in other countries [10]. It also coincided with findings from studies on career choices in China, which showed that family background had a strong influence on students’ job search and that it had a much stronger influence than personal qualifications [23, 31]. In the field of labour economics, researchers referred to the influence of family background on undergraduate students’ job selection as ‘family social capital’, regarding the aspects of economics, culture, social status, and political status [32, 33]. In this research, monthly household income reflected family economic status directly based on a monetary scale. Parents’ education levels directly reflect family cultural background and indirectly reflect family social status from parents’ occupations, for the variable frequently used to represent social status is closely related to education level. Whose opinions were considered most during job selection indirectly reflected family cultural background since it could reveal parents’ educational strategy. Therefore, the results of this research revealed that these three aspects of family capital had significant impacts on pharmacy students’ career intentions.

It was reasonable to position these results within the Chinese cultural context to explain the strong effect of family social capital or family background (including family cultural background, family social status, and family economic situation). However, as some researchers have elucidated, students who felt strong parental expectations might have career dissatisfaction [34]. In recent decades, the conflict between Confucianism-based traditional ethics, and the rapidly developing economy in China has led to an intergenerational contradiction for young people’s career choices [26]. Whether the disharmony between self-gratification and family background will interrupt BS pharmacy students’ career progression and the development of the Chinese pharmacy workforce deserves further attention. More research is needed on this issue.

Finally, this research showed that employment guidance had an impact on the career intention of Chinese pharmacy students. It echoed some studies in western countries which had recommended to establish a comprehensive career support system to help pharmacy students build a clear career perspective in the pharmacy-related field throughout their years of learning [10]. The formation of career intention is a long process that students complete by deploying a variety of occupational information. Pharmacy students have to spend a lot of time in knowledge learning, academic training as well as professional training, thus in a general situation they have fewer opportunities to interact with society and have less information about potential job positions than students in some other majors [35]. On this basis, employment guidance is vital to help them form more real, practical career intentions and have a healthy start in their first jobs [10]. Other research has shown that the transition from school to workplace was the most emotional stage in one’s early career [36]. Although Chinese pharmacy educators had provided a variety of employment guidance, such as lectures, reading materials, and mock interviews [37], pharmacy students still feel unsatisfied [35]. It is time to reform the current collectivistic methods to enhance the personalisation of employment guidance [35]. Moreover, educators’ involvement in employment guidance might balance the aforementioned contradiction between family influence and students’ self-gratification needs.

## Limitations

This preliminary research on the influencing factors of BS pharmacy students’ career intention was conducted in one province in mainland China, which might not be representative of the situation in the whole country. Considering that the NEEP may act as a fence, which might keep students from pursuing a higher-level degree, academic intention was not analysed in this research. The male to female ratio of the sample was less than one-fifth, which was lower than that of the population; this might have introduced gender bias into the analysis. The above limitations could be improved by redesigning the study in the future.

## Conclusion

This research showed that in the Chinese context, the career intention of more than half of the undergraduate pharmacy students was to work in public medical institutions; in other words, they aspired to become hospital pharmacists. In addition, male students were more inclined than female students to quit the pharmacy-related field. Chinese BS pharmacy students’ career intentions were influenced by their self-gratification needs, family backgrounds and the employment guidance. Employment guidance had a positive effect on reserving potential pharmacy workforce. Currently, China is facing an insufficient pharmacy workforce, especially for pharmaceutical care. In addition to knowledge delivery and skills cultivation, educators and policy makers should pay more attention to developing students’ career aspirations, especially by providing efficient employment guidance, according to their gender differences, family backgrounds, and psychological needs. Understanding students’ inclination for self-gratification might help prospective employers design and implement efficient recruitment strategies.

## Supplementary Information

**Additional file 1.** Questionnaire.

## Data Availability

The datasets used and/or analysed during the current study are available from the corresponding author on reasonable request.

## Abbreviations

BS: Bachelor of Science
BPharm: Bachelor of Pharmacy
EFA: Exploratory Factor Analysis
GDP: Gross Domestic Product
KMO: Kaiser-Meyer-Olkin
NEEP: National Entrance Examination for Postgraduate
PharmD: Doctor of Pharmacy
