# Supplementary material for: Final-year pharmacy undergraduate students’ career intention and its influencing factors: a questionnaire study in northwest China
Source: BMC Med Educ. 2020 Nov 4;20:405. doi: 10.1186/s12909-020-02342-8 (PMC7640686; doi:10.1186/s12909-020-02342-8)
Supplement: Supplementary file 1 — Additional file 1. Questionnaire. [file 12909_2020_2342_MOESM1_ESM.docx]

**Questionnaire**

| **Demographic information** | |  | | | | |
| --- | --- | --- | --- | --- | --- | --- |
| D1 | Gender | (A) Male; (B) Female | | | | |
| D2 | Age |  | | | | |
| D3 | Region of origin | (A) Urban; (B) Rural | | | | |
| **Educational situations** | |  | | | | |
| E1 | Study record | (A) Top 30%; (B) Others | | | | |
| E2 | Have you had laboratory training? | (A) Yes; (B) No | | | | |
| E3 | Have you had an internship in any medical institution? | (A) Yes; (B) No | | | | |
| E4 | Have you received employment guidance or not? | (A) Yes; (B) No | | | | |
| **Family Background** | |  | | | | |
| F1 | Father’s education level | (A) Illiterate; (B) Primary; (C) Secondary; (D) Tertiary and above | | | | |
| F2 | Mother’s educational level | (A) Illiterate; (B) Primary; (C) Secondary; (D) Tertiary and above | | | | |
| F3 | Monthly household income per capita (*yuan*) | (A) ~500; (B) 500~999; (C) 1000~1999; (D) 2000~4999; (E) 5000~9999; (F) 10000~ | | | | |
| F4 | Whose opinions were considered most during job selection? (please pick the most ideal one) | (A) Parents; (B) Self; (C) Lover; (D) Friends; (E) Teacher; (F) Others | | | | |
| **Occupational value** | | Very important=5 | Important=4 | Moderately important=3 | Slightly important=2 | Not important=1 |
| I1 | Scale of the institution |  |  |  |  |  |
| I2 | Economic performance of the institution |  |  |  |  |  |
| I3 | Popularity of the institution |  |  |  |  |  |
| I4 | Social image of the institution |  |  |  |  |  |
| I5 | Labour intensity |  |  |  |  |  |
| I6 | Wages and benefits |  |  |  |  |  |
| I7 | Medical equipment |  |  |  |  |  |
| I8 | Display of talents |  |  |  |  |  |
| I9 | Job challenges |  |  |  |  |  |
| I10 | Promotion opportunities |  |  |  |  |  |
| I11 | Interpersonal relationships in the workplace |  |  |  |  |  |
| I12 | Training and self-development opportunities |  |  |  |  |  |
| I13 | Job-major match |  |  |  |  |  |
| I14 | Perspective of the work |  |  |  |  |  |
| I15 | Power of the work |  |  |  |  |  |
| I16 | Significance of the work |  |  |  |  |  |
| I17 | Interest in the work |  |  |  |  |  |
| I18 | Meet family’s expectation |  |  |  |  |  |
| **Career intention** | |  | | | | |
| C0 | What is your career intention?  (please pick the most ideal one) | (A) village clinic; (B) township medical center; (C) county hospital; (D) prefectural hospital; (E) provincial hospital; (F) private medical institute; (G) pharmaceutical industry; (H) official departments on pharmaceutical affairs; (I) community pharmacy; (J) educational institute; (K) other fields | | | | |
